# Chromatogram-Bioactivity Correlation-Based Discovery and Identification of Three Bioactive Compounds Affecting Endothelial Function in Ginkgo Biloba Extract

**DOI:** 10.3390/molecules23051071

**Published:** 2018-05-03

**Authors:** Hong Liu, Li-ping Tan, Xin Huang, Yi-qiu Liao, Wei-jian Zhang, Pei-bo Li, Yong-gang Wang, Wei Peng, Zhong Wu, Wei-wei Su, Hong-liang Yao

**Affiliations:** 1 Guangdong Engineering and Technology Research Center for Quality and Efficacy Re-evaluation of Post-marketed TCM, Guangdong Key Laboratory of Plant Resources, School of Life Sciences, Sun Yat-sen University, 135 Xingangxi Road, Guangzhou 510275, China; beauty19880711@163.com (H.L.); tanliping2017@126.com (L.-p.T.); huangxin1989@126.com (X.H.); liaoyiqiu@aliyun.com (Y.-q.L.); zhweij6@mail2.sysu.edu.cn (W.-j.Z.); lipb73@126.com (P.-b.L.); awad7476@163.com (Y.-g.W.); pweiyu929@126.com (W.P.); wuzhong1962@126.com (Z.W.); lsssww@126.com (W.-w.S.); 2 Medical College, Shaoguan University, 1 Xinhuanan Road, Shaoguan 512026, China

**Keywords:** *Ginkgo biloba* Extract (GBE), chromatogram-bioactivity correlation, bioactive compounds, endothelial function

## Abstract

Discovery and identification of three bioactive compounds affecting endothelial function in *Ginkgo biloba* Extract (GBE) based on chromatogram-bioactivity correlation analysis. Three portions were separated from GBE via D101 macroporous resin and then re-combined to prepare nine GBE samples. 21 compounds in GBE samples were identified through UFLC-DAD-Q-TOF-MS/MS. Correlation analysis between compounds differences and endothelin-1 (ET-1) in vivo in nine GBE samples was conducted. The analysis results indicated that three bioactive compounds had close relevance to ET-1: Kaempferol-3-*O*-α-l-glucoside, 3-*O*-{2-*O*-{6-*O*-[P-OH-trans-cinnamoyl]-β-d-glucosyl}-α-rhamnosyl} Quercetin isomers, and 3-*O*-{2-*O*-{6-*O*-[P-OH-trans-cinnamoyl]-β-d-glucosyl}-α-rhamnosyl} Kaempferide. The discovery of bioactive compounds could provide references for the quality control and novel pharmaceuticals development of GRE. The present work proposes a feasible chromatogram-bioactivity correlation based approach to discover the compounds and define their bioactivities for the complex multi-component systems.

## 1. Introduction

**Figure 1:**
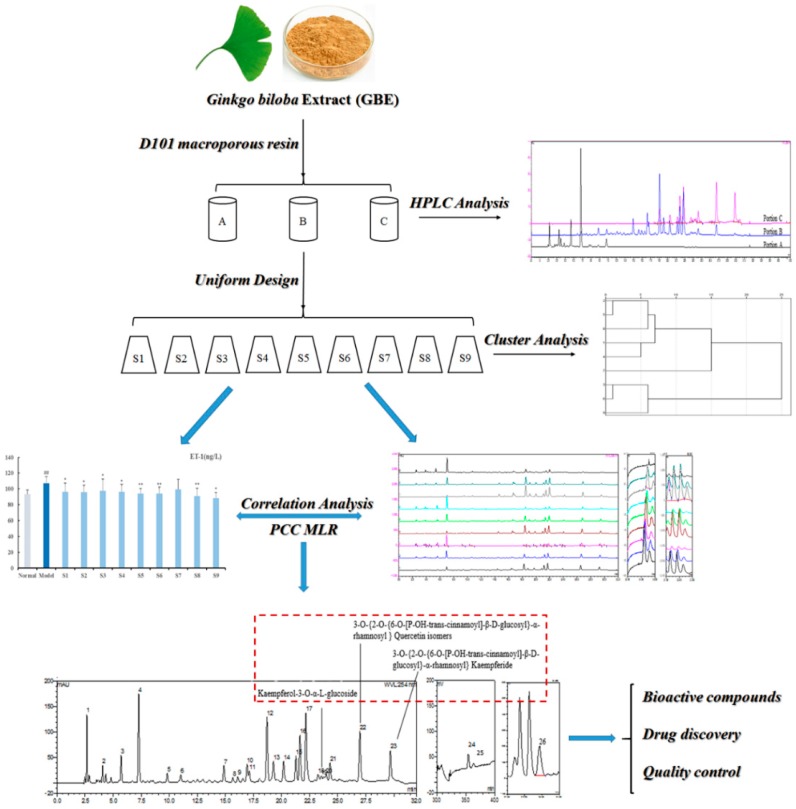
Research process for discovery of bioactive compounds affecting endothelial function in GBE.

*Ginkgo biloba* Extract (GBE), extracted from *Ginkgo biloba* leaves, is mainly composed of terpene trilactones, flavonoid heterosides, ginkgolic acids, phenolic acids, proanthocyanidins, etc. [1,2]. GBE can significantly decrease serum ET-1 to reverse endothelial dysfunction [3,4,5]. Nowadays, chromatographic fingerprint plays a vital role in the quality control of GBE, including for authenticity determination and chemical information analyses. However, existing GBE studies with fingerprint tech mainly focus on the chemical characteristics, but do not elaborate the correlation between compounds and their bioactive effects. Based on the hypothesis that bioactive effects varied with differences between compounds, chromatographic fingerprint and bioactive tests of nine re-combined GBE samples were conducted, and their correlations were further analyzed (Figure 1). Other than the usual methods of isolation, purification, and then biotests, this study provided a feasible approach for exploring the bioactive compounds in complex systems.

## 2. Results

### 2.1. GBE HPLC Fingerprint and Identification of Components

**Figure 2:**
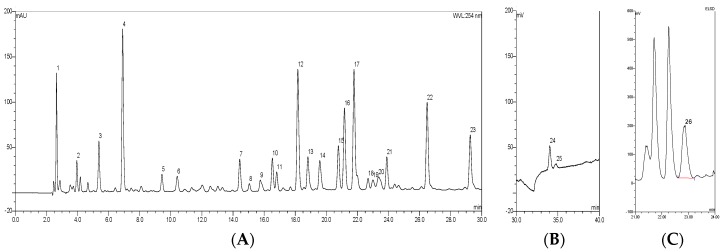
The HPLC fingerprint of GBE with UV (**A**) and ELSD (**B**,**C**).

With optimized HPLC conditions, the standard GBE HPLC fingerprint (Figure 2) was established, and 21 compounds were identified or characterized through the HPLC-DAD-ELSD-MS/MS technique in our previous work [6] (Table 1). According to the retention time, UV spectra, and MS spectra of the reference standards, Protocatechuic acid (P_4_), Rutin (P_12_), Ginkgolide A (P_24_), Ginkgolide B (P_25_), and Bilobalide (P_26_) were identified unambiguously. The other compounds were characterized according to MS fragmentation pattern, UV spectra, and the reported literature.

### 2.2. Three Portions Separated from GBE and Nine Re-Combined GBE Samples

**Figure 3:**
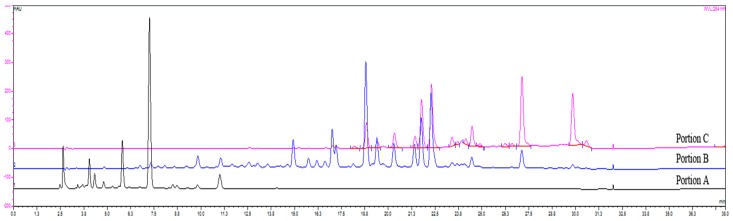
The HPLC fingerprints of three portions separated from GBE via D101 macroporous resin.

**Figure 4:**
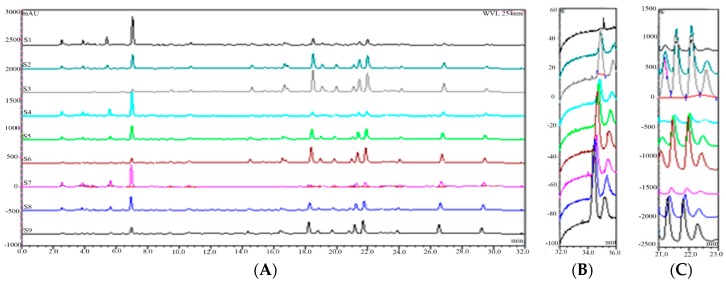
The HPLC fingerprints of nine GBE samples with UV (**A**) and ELSD (**B**,**C**).

Portion A, portion B, and portion C were separated from GBE via D101 macroporous resin. They were re-combined with different compositions to get the nine GBE samples (Figure 3). In accordance with the optimized HPLC conditions, the HPLC fingerprints of the nine GBE samples (S_1_–S_9_) were constructed (Figure 4). 26 peak areas in nine GBE samples are shown in Table 2.

### 2.3. Cluster Analysis of Nine GBE Samples

**Figure 5:**
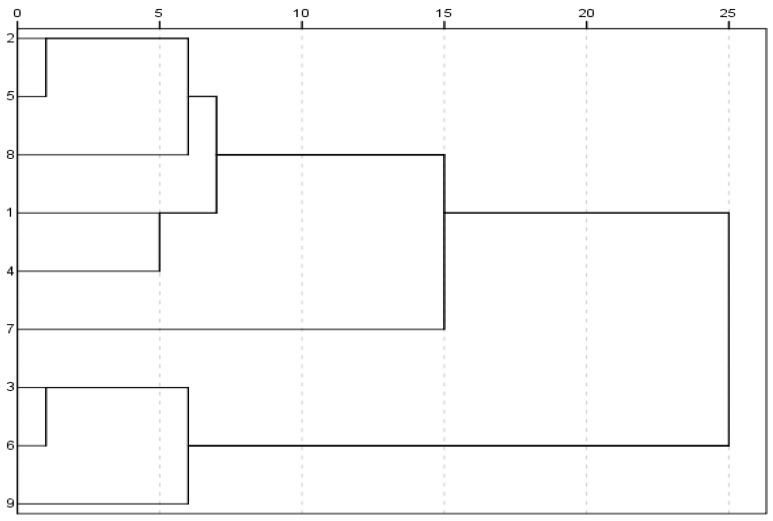
The dendrogram of cluster analysis of the nine GBE samples.

Based on the data of the 26 peak areas, Cluster analysis was performed in SPSS 19.0. The clustering method was Nearest Neighbor. The distance calculation method was Euclidean Distance. The rescaled distance cluster combine was defined as 5. Nine GBE samples could be divided into seven categories (Figure 5): S_2_ and S_5_ belonged to a class, S_3_ and S_6_ belonged to a class, and the remaining samples respectively represented a class each. Cluster analysis results indicated that the nine GBE samples had chemical differences in their compounds.

### 2.4. ET-1 Biotests of Nine GBE Samples

Plasma ET-1 in vivo was detected in the 11 treatment groups (Table 3). Compared with the normal group, plasma ET-1 content significantly increased in the model group. Compared with the model group, plasma ET-1 content significantly decreased in the S1, S2, S3, S4, S5, S6, S8, and S9 groups, but not for the S7 group. Biotest results indicated that nine GBE samples showed biological differences for ET-1.

### 2.5. CA between Compound Differences and Biological Differences

Dimensionless data of the peak areas of 26 compounds and their ET-1 values are shown in Table S1. The Pearson correlation coefficients (PCC) are shown in Table 4. The results indicated that P_18_, P_22_, and P_23_ had a significantly positive relation with ET-1, but that P_1_, P_2_, P_3_, P_4_, and P_6_ were negatively correlated to ET-1.

The scores of the extracted C1 and C2 were used as the new independent variables (Table 5). The strict regression equation between C1, C2 and ET-1 was established as follows: ET-1 = 94.68 + 0.678 × C1 + 2.626 × C2 (R = 0.801, Sig. < 0.05).

In accordance with the rotated component matrix (Table S3), C1 and C2 were replaced by the 26 original independent variables (P_1_–P_26_). Regression coefficients (RC) of P_1_–P_26_ are shown in Table 6. The results were in accordance with the PC analysis, indicating that P_18_, P_20_, P_22_, P_23_, and P_24_ had a highly positive relation with ET-1, but that P_1_, P_2_, P_3_, P_4_, and P_6_ showed a negative correlation.

## 3. Discussion

Current research methods for natural medicine mainly fall into two directions. The first is to separate single components or the active part and then assess the biological effects in vivo or in vitro; the second is to match up the compounds and bio-effects in the whole herb using computational modelling. It is understood that separating and assessing each compound one by one is almost impossible. Numerous existing studies of GBE focus on the chemical identification and the biological effects, separately, but not the correlation between them. ET-1 is a potent vasoconstrictor peptide released from endothelial cells [7]. Several studies have demonstrated that exposure to cold is associated with raised plasma ET-1 [8,9]. Thus, a rat model combined with subcutaneous injection of adrenaline and ice-bath was established, and similar data was observed in the present study.

GBE’s main bioactive constituents include flavonoid glycosides and terpene trilactones. Flavonoid glycosides were detected by HPLC-UV [10,11,12]. Terpene trilactones were detected by Evaporative Light Scattering Detector (ELSD) due to their poor UV absorption property. Thus, GBE’s chromatographic fingerprint was established by HPLC-UV-ELSD, in which 21 compounds were identified or characterized through the UFLC-DAD-Q-TOF-MS/MS technique. To prepare appropriate GBE samples with varying compounds, three portions were separated from GBE using D101 macroporous resin, and then re-combined to get nine GBE samples. The different ratios of the three portions were designed using a four-factor, nine-level Uniform Design (UD) method, which has been successfully applied to prepare different Chinese medicine samples [13,14]. To guarantee the differences of the GBE samples, cluster analysis was conducted that nine GBE samples could be divided into seven categories.

**Figure 6:**
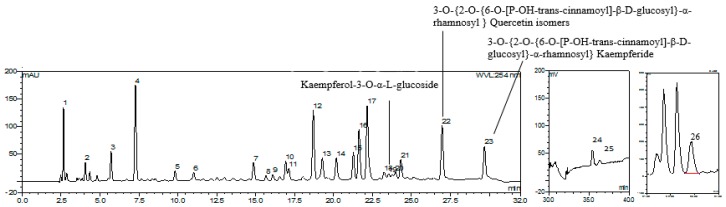
Three core bioactive compounds of GBE related to ET-1.

Correlation analysis was applied to discover and predict the compounds with bioactivities in our previous work [15,16]. The discovery of bioactive compounds was based on the hypothesis that the effect varies based on differences in the compounds. If a compound varies a little, while showing a big difference in the effect, the compound will be considered to have a close relevance; in the opposite case, the compound will be considered to have no effect contribution. In the cluster analysis, although S2 and S5, S3 and S6 belonged to a class, there were still relatively large differences among the discovered bioactive compounds, and this might be the reason behind the differences in effect among them. In this work, the Pearson Correlation and Multiple Linear Regression methods were used to evaluate the effect contribution of each compound, and the analysis results of the two methods were highly consistent. The connections between the identified compounds and ET-1 are presented dynamically in the electronic supplementary material (Compound-effect bubble chart). Kaempferol-3-*O*-α-l-glucoside (P_18_), 3-*O*-{2-*O*-{6-*O*-[P-OH-trans-cinnamoyl]-β-d-glucosyl}-α-rhamnosyl} Quercetin isomers (P_22_), and 3-*O*-{2-*O*-{6-*O*-[P-OH-trans-cinnamoyl]-β-d-glucosyl}-α-rhamnosyl} Kaempferide (P_23_) were significantly correlated to ET-1 (Figure 6). Numerous preclinical studies provide support for flavonoids exhibiting protective effects on endothelial dysfunction [17]. Quercetin, modified from quercetin flavonoid during metabolism, inhibits the overproduction and gene expression of ET-1 in vitro [18,19]. Kaempferol can improve the endothelial damage [20], but there is no direct evidence for either Kaempferol and Kaempferide on regulating ET-1. In GBE, not all the flavonoid glycosides have strong inhibitory activity on ET-1 release. As for terpene trilactones in GBE, Ginkgolide A and Ginkgolide B had a highly positive correlation, which also contributed to the effects. Moreover, P_1_, P_2_, P_3_, P_4_, and P_6_ from portion A were negatively correlated with ET-1. Despite having no statistical meaning, the results suggested that water-soluble constituents might induce endothelial dysfunction, but this needs further experiments to confirm.

## 4. Materials and Methods

### 4.1. Animals and Materials

Sprague-Dawley male rats, Specific pathogen-free, 250–300 g, were purchased from Guangdong Medical Laboratory Animal Center (SCXK-(Yue) 2013-0002). Rats were fed on standard laboratory diet and water and kept in environmentally controlled quarters with temperature maintained at 25 °C and a 12 h dark-light cycle for a week before use. Experiments were approved by the Animal Care and Use Committee of Sun Yat-sen University (2015062529) and performed in accordance with guidelines of Institutional Animal Care and Use Committee for U.S. institutions. GBE was manufactured by INDENA S.P.A (batch: 15271). GBE Injection, the sterile solution of GBE, was purchased from Yue Kang Pharmaceutical Group Co., Ltd. (batch: 05121108) (Beijing, China). Adrenalin (Adr) Hydrochloride Injection was purchased from Yuanda Medical (Harbin, China) Co., Ltd. (batch: 150412). 1,2-propanediol and absolute ethyl alcohol was purchased from Tianjin Fuyu Chemical Co., Ltd. (batch: 20141026) (Tianjin, China). Rat ET-1 Elisa Assay Kit was purchased from Nanjing Jiancheng Bioengineering Institute. D101 macroporous resin was purchased from Xi’an Butian Adsorption Materials Co., Ltd. (batch: 20140918) (Xi’an, China).

### 4.2. Preparation of GBE Samples

GBE (315 mg) was separated into three portions via D101 macroporous resin (20 g), with the eluent of 550 mL purified water (Portion A); 100 mL ethanol (40%, *v*/*v*, Portion B), and 100 mL absolute ethyl alcohol (Portion C). Each portion was evaporated with a rotary evaporator and dissolved in 1,2-propanediol (25%, g/mL) to 30 mL for HPLC analysis. According to a four-factor, nine-level UD (Table 7), three portions were re-combined to get nine GBE samples. GBE Samples were stored at 4 °C before use.

### 4.3. HPLC Fingerprint and Cluster Analysis

GBE analyses were performed on an UltiMate 3000 series Dual-Gradient Analytical LC System (Dionex, Thermo Fisher Scientific Inc., Waltham, MA, USA), equipped with DAD and ELSD. The HPLC-DAD-ELSD conditions were as follows [6]: Chromatographic separation was carried out using an Agilent zorbax SB C18 column (4.6 mm × 250 mm, 5 μm) as an analytical column and a Dionex Acclaim Polar Advantage C18 column (3.0 mm × 50 mm, 3 μm) as a pretreatment column, and operated at 25 °C; Mobile phase consisted of acetonitrile (A), tetrahydrofuran (B), formic acid (C, 0.1%, *v*/*v*) with a multi-step gradient elution (A: 0–27 min: 10%→28%, 27–27.1 min: 28%→1%, 27.1–40 min: 1%→25%; B: 0–27 min: 0%→0%, 27–27.1 min: 0%→15%, 27.1–40 min: 15%→15%; C: 0–27 min: 90%→78%, 27–27.1 min: 72%→84%, 27.1–40 min: 84%→60%) at a flow rate of 1.0 mL/min; Drift tube temperature of ELSD was set at 50 °C, and the nebulizing gas pressure was 3.5 bar with a gain value of 11; Sample volume was set at 10 μL. Data were controlled by Chromeleon 6.8 chromatography data system. 26 peak areas in the HPLC fingerprint were used for Cluster Analysis in SPSS 19.0 (IBM, Armonk, NY, USA). The clustering method was Nearest Neighbor, and the distance calculation method was Euclidean Distance. The rescaled distance cluster combine was set at 5.

### 4.4. Modelling and ET-1 Assay

Rats were randomly divided into eleven groups of normal (normal saline: NS, 7.2 mL/kg, *n* = 10) as blank, model (NS, 7.2 mL/kg, *n* = 10) as negative control, and nine GBE samples (7.2 mL/kg, *n* = 10), receiving intraperitoneal injection once daily for 7 consecutive days. After the 7th administration, the rats—except those in normal group—were subcutaneously injected with Adr (0.8 mg/kg). After 2 h, rats were kept in ice-water (0–2 °C) for 4 min, and 2 h later were subcutaneously re-injected with Adr (0.8 mg/kg). All the rats were fasted for 12 h. Blood was collected through abdominal aortic. Plasma ET-1 was detected by Elisa kit.

### 4.5. Correlation Analysis between Compound Difference and Bioactivity Difference

*Pearson Correlation*. 26 Peak areas were regarded as independent variables (P_1_–P_26_). Average ET-1 value was regarded as a dependent variable. Every value of the peak areas and ET-1 in Table 2 was divided by the average of each column to get dimensionless data (Table S1). Pearson Correlation was used to analyze the correlation among P_1_–P_26_ and ET-1. *Multiple Linear Regression*. 26 independent variables (P_1_–P_26_) were recombined into two mutual independent principal components, which were regarded as new independent variables (C1 and C2, contributing to 96.388% of the total variance, Table S2). The regression equation between two components (C1 and C2) and ET-1 parameter was constructed by a stepwise regression analysis approach. Once a strict regression equation was established (*p* < 0.05), C1 and C2 would be replaced by the 26 original independent variables (P_1_–P_26_) according to the rotated component matrix (Table S3). Then, the regression coefficients of P_1_–P_26_ were used to evaluate the effect contribution.

### 4.6. Statistical Analysis

Experimental data were presented as mean ± standard deviation and analyzed by One-Way Analysis of Variance. *p*-values less than 0.05 or 0.01 were considered statistically significant.

## 5. Conclusions

Kaempferol-3-*O*-α-l-glucoside, 3-*O*-{2-*O*-{6-*O*-[P-OH-trans-cinnamoyl]-β-d-glucosyl}-α-rhamnosyl} Quercetin isomers, and 3-*O*-{2-*O*-{6-*O*-[P-OH-trans-cinnamoyl]-β-d-glucosyl}-α-rhamnosyl} Kaempferide were discovered to have the closest relevance to ET-1, which has not been reported so far and could provide further reference for the quality control and novel pharmaceutical development of GRE. Moreover, this work proposes a feasible approach for the discovery and prediction of compounds and their bioactivities in complex systems, especially for traditional Chinese medicine. The specific process is as follows: prepare the samples by the re-combination of different parts; establish the HPLC fingerprints; evaluate the bio-effects in vivo; regard the compound differences and effect differences as mathematical variables; analyze the relevance between the variables to find key bioactive compounds.

## Figures and Tables

**Table 1:** Identification of 21 compounds in GBE HPLC fingerprint by UFLC-DAD-Q-TOF-MS/MS.

Peaks	Retention Time	Major Fragment Ions (MS/MS)	Identified Compounds
P_1_	2.520		-
P_2_	3.840		-
P_3_	5.670		-
P_4_	6.960	137.0235 [M + H-H_2_O]^+^, 109.028 [M + H-H_2_O-CO]^+^, 93.0348 [M + H-H_2_O-CO_2_]^+^	Protocatechuic acid ^a^
P_5_	9.403		-
P_6_	10.617		-
P_7_	14.367	611.1586 [M + H-rha]^+^, 465.1014 [M + H-2rha]^+^, 303.0496 [M + H-2rha-glu]^+^	3-*O*-[2-*O*,6-*O*-double(α-l-rhamnosyl)-β-d-glucosyl] Quercetin
P_8_	15.207	319.0444 [M + H-rha-glu]^+^	3-*O*-[6-*O*-(α-l-rhamnosyl)-β-d-glucosyl] Myricetin
P_9_	15.607	319.0454 [M + H-glu]^+^	3-*O*-[β-d-glucosyl] Myricetin
P_10_	16.420	595.1643 [M + H-rha]^+^, 449.1073 [M + H-2rha]^+^, 287.0552 [M + H-2rha-glu]^+^	3-*O*-[2-*O*,6-*O*-double(α-l-rhamnosyl)-β-d-glucosyl] Kaempferide
P_11_	16.613	625.174 [M + H-rha]^+^, 479.1167 [M + H-2rha]^+^, 317.0650 [M + H-2rha-glu]^+^,	3-*O*-[2-*O*,6-*O*-double(α-l-rhamnosyl)-β-d-glucosyl] Isorhamnetin
P_12_	18.233	465.1012 [M + H-rha]^+^ 303.0496 [M + H-rha-glu]^+^,	3-*O*-[6-*O*-(α-l-rhamnosyl)-β-d-glucosyl] Quercetin (rutin) ^a^
P_13_	18.813	495.1122 [M + H-rha]^+^, 333.0600 [M + H-glu-rha]^+^	3-*O*-[6-*O*-(α-l-rhamnosyl)-d-glucosyl] Queretagetin
P_14_	19.720	303.0501 [M + H-glu]^+^	Quercetin-3-*O*-β-d-glucoside
P_15_	20.807	303.0501 [M + H-rha-glu]^+^	3-*O*-[2-*O*-(β-d-glucosyl)-α-l-rhamnosyl] Quercetin
P_16_	21.173	287.0546 [M + H-rha-glu]^+^	3-*O*-[6-*O*-(β-d-glucosyl)-α-l-rhamnosyl] Kaempferide
P_17_	21.693	479.1176 [M + H-rha]^+^, 317.0658 [M + H-rha-glu]^+^	3-*O*-[6-*O*-(β-d-glucosyl)-α-l-rhamnosyl] Isorhamnetin
P_18_	22.790	287.055 [M + H-glu]^+^	Kaempferol-3-*O*-α-l-glucoside
P_19_	23.057	347.0761 [M + H-rha-glu] +	3-*O*-[6-*O*-(α-l-rhamnosyl)-β-d-glucosyl] Syringetin
P_20_	23.487	347.0767 [M + H-rha-glu] +	3-*O*-[2-*O*-(α-l-rhamnosyl)-β-d-glucosyl] Syringetin
P_21_	23.867	287.0569 [M + H-rha-glu]^+^	3-*O*-[2-*O*-(β-d-glucosyl)-α-l-rhamnosyl] Kaempferide
P_22_	26.527	449.101 [M + H-rha-glu]^+^,	3-*O*-{2-*O*-{6-*O*-[P-OH-trans-cinnamoyl]-β-d-glucosyl}-α-rhamnosyl} Quercetin isomers
P_23_	29.233	433.1063 [M + H-rha-glu]^+^,	3-*O*-{2-*O*-{6-*O*-[P-OH-trans-cinnamoyl]-β-d-glucosyl}-α-rhamnosyl} Kaempferide
P_24_	34.379	391.1396 [M + H-H_2_O]^+^; 373.1075 [M + H-2H_2_O]^+^, 345.13 [M + H-2H_2_O-CO]^+^,	Ginkgolide A ^a^
P_25_	35.195	407.1368 [M + H-H_2_O]^+^, 389.1262 [M + H-2H_2_O]^+^, 361.1304 [M + H-2H_2_O-CO]^+^,	Ginkgolide B ^a^
P_26_	22.296	309.3054 [M + H-H_2_O]^+^	Bilobalide ^a^

^a^ Identification in comparison with reference standards.

**Table 2:** The 26 peak areas of the nine GBE samples.

Samples	P_1_	P_2_	P_3_	P_4_	P_5_	P_6_	P_7_	P_8_	P_9_	P_10_	P_11_	P_12_	P_13_
S_1_	1.2622	1.3788	2.1911	12.1766	2.8357	3.524	6.5859	2.0551	1.4858	8.3926	5.1951	28.4887	7.0591
S_2_	3.1069	3.3891	5.7438	26.3239	2.0025	3.6599	3.8695	1.1924	0.801	4.7887	3.0219	17.4548	4.1193
S_3_	5.1957	5.5726	9.7592	42.5976	1.0266	3.213	0	0	0	0	0	4.356	1.0038
S_4_	0	0	0	9.8952	3.9214	4.981	9.2285	2.8734	2.0094	11.422	6.9621	37.5392	8.7436
S_5_	3.2118	3.5892	5.5843	27.3079	2.991	4.6368	5.9836	1.8489	1.3209	7.3954	4.4638	24.571	5.7354
S_6_	7.4305	6.3609	10.8327	48.0321	1.7808	4.3221	0	0	0	0	0	8.5261	1.9302
S_7_	0	0	0	6.0996	5.5958	5.3295	12.8322	4.5302	3.3714	17.5349	10.0728	52.8165	12.5493
S_8_	3.5617	3.7739	6.3758	28.8564	4.426	5.4215	9.0171	3.2387	2.2417	11.871	6.9002	36.2443	8.6628
S_9_	9.3721	7.5017	13.1006	57.1505	3.0606	5.2422	4.1496	1.4944	1.0327	5.3503	3.1294	15.863	3.8046
**Samples**	**P_14_**	**P_15_**	**P_16_**	**P_17_**	**P_18_**	**P_19_**	**P_20_**	**P_21_**	**P_22_**	**P_23_**	**P_24_**	**P_25_**	**P_26_**
S_1_	9.4772	8.1063	23.5872	35.2178	3.0655	0.6415	1.9176	8.2878	25.8635	15.4303	4.9291	1.8927	18.2607
S_2_	6.4263	5.2957	16.7922	24.4355	2.3935	0.4798	1.5539	5.2362	19.9002	12.9111	3.7011	1.1571	13.0276
S_3_	2.6031	1.9266	8.5073	11.4559	1.587	0	1.0682	3.2834	14.0843	10.6601	5.6539	1.8087	7.1442
S_4_	11.5354	10.171	27.3343	41.7826	3.0651	0.5678	1.9455	8.7336	25.4447	14.135	2.9817	0.4018	19.4095
S_5_	7.8271	6.8175	18.9692	28.6363	2.2589	0.4425	1.4746	5.2605	17.9486	10.0593	2.0966	0.1254	10.8716
S_6_	3.2396	2.6844	8.7539	12.6152	1.2919	0	0	2.8117	10.7481	7.2251	0	0	6.5011
S_7_	15.3825	13.8241	33.6609	52.7184	3.4513	0.7236	2.1981	9.7774	25.1843	10.9571	2.0016	0.3504	23.2875
S_8_	10.5258	9.4589	22.7495	35.6553	2.3213	0.5586	1.443	5.3602	16.1803	5.931	0.7501	0.3917	14.6142
S_9_	4.4374	4.0615	9.2461	14.7265	0	0	0	2.0158	5.1178	1.4163	0	0	5.2694

**Table 3:** The content of ET-1 in plasma.

Group	ET-1 (ng/L)
Normal	93.07 ± 5.45
Model	107.07 ± 8.50 ^##^
S1	96.15 ± 11.45 *
S2	95.72 ± 8.88 *
S3	97.40 ± 15.21 *
S4	96.30 ± 9.68 *
S5	93.89 ± 6.76 **
S6	94.16 ± 8.49 **
S7	99.37 ± 12.65
S8	90.82 ± 10.19 **
S9	88.31 ± 7.19 *

^##^*p* < 0.01 when compared with normal. * *p* < 0.05 and ** *p* < 0.01 when compared with model.

**Table 4:** PCCs between 26 components and ET-1.

Variables	PCC	Variables	PCC	Variables	PCC
P1	−0.598	P10	0.209	P19	0.406
P2	−0.658	P11	0.214	P20	0.647
P3	−0.651	P12	0.277	P21	0.635
P4	−0.658	P13	0.273	P22	0.731 *
P5	0.046	P14	0.365	P23	0.806 **
P6	−0.414	P15	0.332	P24	0.652
P7	0.198	P16	0.461	P25	0.474
P8	0.167	P17	0.424	P26	0.577
P9	0.198	P18	0.727 *		

Note: * *p* < 0.05 and ** *p* < 0.01.

**Table 5:** The scores of two components C1 and C2.

Samples	C1	C2
S_1_	0.055	1.411
S_2_	−0.501	0.768
S_3_	−1.484	0.772
S_4_	0.828	0.533
S_5_	0.055	−0.118
S_6_	−1.011	−0.770
S_7_	1.739	0.011
S_8_	0.775	−0.787
S_9_	−0.456	−1.820

**Table 6:** RC between 26 components and ET-1 (Model Sig. < 0.05).

Variables	RC	Variables	RC	Variables	RC
P_1_	−2.297	P_10_	0.777	P_19_	1.779
P_2_	−2.127	P_11_	0.851	P_20_	2.292
P_3_	−2.127	P_12_	0.902	P_21_	2.019
P_4_	−2.202	P_13_	0.921	P_22_	2.445
P_5_	0.127	P_14_	1.196	P_23_	2.637
P_6_	−1.362	P_15_	1.081	P_24_	2.259
P_7_	0.822	P_16_	1.522	P_25_	1.913
P_8_	0.649	P_17_	1.395	P_26_	1.860
P_9_	0.675	P_18_	2.291		

**Table 7:** Volumes and percentage of three portions in nine GBE samples.

Sample	Portion A mL (% ^a^)	Portion B mL (%)	Portion C mL (%)
S_1_	2.50 (50)	7.50 (150)	10.00 (200)
S_2_	6.25 (125)	3.75 (75)	8.75 (175)
S_3_	10.00 (200)	0 (0)	7.50 (150)
S_4_	1.25 (25)	8.75 (175)	6.25 (125)
S_5_	5.00 (100)	5.00 (100)	5.00 (100)
S_6_	8.75 (175)	1.25 (25)	3.75 (75)
S_7_	0 (0)	10.00 (200)	2.50 (50)
S_8_	3.75 (175)	6.25 (125)	1.25 (25)
S_9_	7.50 (150)	2.50 (50)	0 (0)

Note: ^a^ % represents the nine levels (0, 25%, 50%, 75%, 100%, 125%, 150%, 175%, 200%) of each portion A, B, C, and the sequence was designed according to a four-factor, nine-level UD method.

## Supplementary Materials

The following are available online at http://www.mdpi.com/1420-3049/23/5/1071/s1, Table S1: The dimensionless data of 26 peak areas and parameter ET-1 values; Table S2: The total variance explained of two Components; Table S3: The rotated component matrix; Compound-effect bubble chart.

## Abbreviations

GBE	Ginkgo biloba Extract
CA	Correlation analysis
ET-1	endothelin-1
PCC	Pearson correlation coefficients
RC	Regression coefficients
ELSD	Evaporative Light Scattering Detector
UD	Uniform Design
Adr	Adrenalin
