# Supplementary material for: Chromatogram-Bioactivity Correlation-Based Discovery and Identification of Three Bioactive Compounds Affecting Endothelial Function in Ginkgo Biloba Extract
Source: Molecules. 2018 May 3;23(5):1071. doi: 10.3390/molecules23051071 (PMC6102599; doi:10.3390/molecules23051071)
Supplement: Supplementary file 1 [file molecules-23-01071-s001.pdf]

Supplementary Materials

Table S1-S3

Table S1. The dimensionless data of 26 peak areas and parameter ET-1 values

Samples	P_1	P_2	P_3	P_4	P_5	P_6	P_7	P_8	P_9	P_{10}	P_{11}	P12	P_{13}
S_1	0.3427	0.3931	0.3679	0.4240	0.9233	0.7864	1.1472	1.0732	1.0904	1.1315	1.1763	1.1352	1.1851
S_2	0.8437	0.9662	0.9646	0.9167	0.6520	0.8167	0.6740	0.6227	0.5878	0.6456	0.6842	0.6955	0.6915
S_3	1.4109	1.5888	1.6390	1.4834	0.3342	0.7170	0.0000	0.0000	0.0000	0.0000	0.0000	0.1735	0.1685
S_4	0.0000	0.0000	0.0000	0.3445	1.2768	1.1115	1.6075	1.5006	1.4747	1.5399	1.5765	1.4958	1.4679
S_5	0.8722	1.0233	0.9378	0.9509	0.9739	1.0347	1.0423	0.9655	0.9694	0.9970	1.0107	0.9790	0.9628
S_6	2.0178	1.8135	1.8193	1.6726	0.5798	0.9645	0.0000	0.0000	0.0000	0.0000	0.0000	0.3397	0.3240
S_7	0.0000	0.0000	0.0000	0.2124	1.8220	1.1893	2.2352	2.3659	2.4743	2.3640	2.2809	2.1046	2.1068
S_8	0.9672	1.0759	1.0708	1.0049	1.4411	1.2098	1.5707	1.6914	1.6452	1.6004	1.5624	1.4442	1.4543
S_9	2.5451	2.1388	2.2002	1.9902	0.9965	1.1698	0.7228	0.7804	0.7579	0.7213	0.7086	0.6321	0.6387

Samples	P_{14}	P_{15}	P_{16}	P_{17}	P_{18}	P_{19}	P_{20}	P_{21}	P_{22}	P_{23}	P_{24}	P_{25}	P_{26}	ET-1
S_1	1.1936	1.1701	1.2516	1.2321	1.4196	1.6912	1.4876	1.4692	1.4505	1.5651	2.0060	2.7798	1.3882	1.0155
S_2	0.8094	0.7644	0.8910	0.8549	1.1084	1.2649	1.2055	0.9282	1.1160	1.3096	1.5063	1.6995	0.9904	1.011
S_3	0.3278	0.2781	0.4514	0.4007	0.7349	0.0000	0.8287	0.5820	0.7899	1.0813	2.3010	2.6565	0.5431	1.0287
S_4	1.4529	1.4682	1.4505	1.4618	1.4194	1.4969	1.5093	1.5483	1.4270	1.4338	1.2135	0.5901	1.4756	1.0171
S_5	0.9858	0.9841	1.0066	1.0018	1.0460	1.1665	1.1439	0.9325	1.0066	1.0203	0.8533	0.1842	0.8265	0.9917
S_6	0.4080	0.3875	0.4645	0.4413	0.5982	0.0000	0.0000	0.4984	0.6028	0.7328	0.0000	0.0000	0.4942	0.9945
S_7	1.9374	1.9955	1.7862	1.8444	1.5982	1.9076	1.7052	1.7333	1.4124	1.1114	0.8146	0.5146	1.7704	1.0495
S_8	1.3257	1.3654	1.2072	1.2474	1.0749	1.4726	1.1194	0.9502	0.9074	0.6016	0.3053	0.5753	1.1110	0.9592
S_9	0.5589	0.5863	0.4906	0.5152	0.0000	0.0000	0.0000	0.3573	0.2870	0.1436	0.0000	0.0000	0.4006	0.9327

Table S2. The total variance explained of two Components.

Component	variance %	Accumulated variance %
C1	76.182	76.182
C2	20.206	96.388

Table S3. The rotated component matrix.

Peaks	C1	C2
P1	-0.704	-0.693
P2	-0.775	-0.610
P3	-0.773	-0.610
P4	-0.758	-0.643
P5	0.973	-0.203
P6	0.694	-0.698
P7	0.989	0.058
P8	0.987	-0.008

P9	0.987	0.002
P10	0.990	0.040
P11	0.989	0.069
P12	0.995	0.087
P13	0.992	0.095
P14	0.978	0.203
P15	0.986	0.157
P16	0.941	0.336
P17	0.958	0.284
P18	0.675	0.698
P19	0.837	0.461
P20	0.681	0.697
P21	0.811	0.560
P22	0.625	0.770
P23	0.186	0.956
P24	-0.203	0.913
P25	-0.319	0.811
P26	0.861	0.486

Electronic Supplementary Material

Compound-effect bubble chart.swf
